# An alternating multilayer architecture boosts ultrahigh energy density and high discharge efficiency in polymer composites†

**DOI:** 10.1039/c9ra10030j

**Published:** 2020-02-06

**Authors:** Tao Zhang, Zhenkang Dan, Zhonghui Shen, Jianyong Jiang, Mengfan Guo, Bin Chen, Yuanhua Lin, Ce-Wen Nan, Yang Shen

**Affiliations:** School of Materials Science and Engineering, State Key Lab of New Ceramics and Fine Processing, Tsinghua University Beijing 100084 China shyang_mse@tsinghua.edu.cn chenbinalvin@mail.tsinghua.edu.cn

## Abstract

Poly(vinylidene fluoride) (PVDF)-based polymers with excellent flexibility and relatively high permittivity are desirable compared to the traditional bulk ceramic in dielectric material applications. However, the low discharge efficiency (<70%) caused by the severe intrinsic dielectric loss of these polymers result in a decrease in their breakdown strength and other problems, which limit their widespread applications. To address these outstanding issues, herein, we used a stacking method to combine poly(methyl methacrylate) (PMMA) with poly(vinylidene fluoride-*co*-hexafluoropropylene) (P(VDF-HFP)) for the synthesis of a series of alternating multilayer films with different layers. Benefitting from the blocking effect of the multilayer structure and excellent insulation performance of PMMA, simultaneous improvements in the breakdown strength and discharge efficiency of the multilayer films were achieved. Compared with the pure polymer films and other multilayer films with different layers, the film with a 9-layer structure exhibited the highest energy storage density of 25.3 J cm^−3^ and extremely high discharge efficiency of 84% at 728 MV m^−1^. Moreover, the charge and discharge performance of the other multilayer films were also better than that of P(VDF-HFP). In addition, it was also found that for the multilayer composite films with the same components, the blocking effect was reinforced with an increase in the number of layers, which led to a significant improvement in the breakdown strength. We consider that the multilayer structure can correlate with the dielectric properties of different polymer materials to enhance the energy storage of composite materials, and will provide a promising route to design high dielectric performance devices.

## Abstract 2

Poly(vinylidene fluoride) (PVDF)-based polymers with excellent flexibility and high breakdown strengths are desirable compared to the traditional bulk ceramic in dielectric material applications.

## Graphical abstract

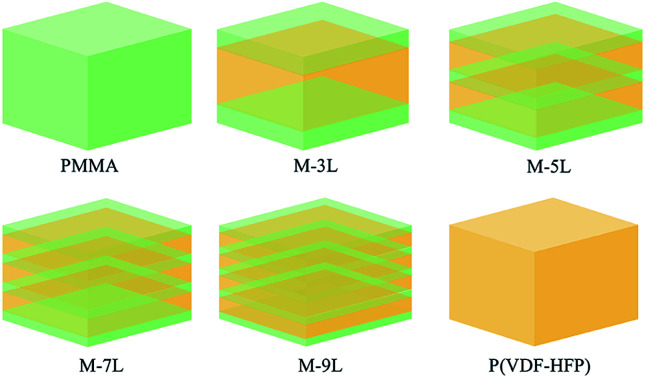

## Introduction

The rapid development of electronic technology worldwide has resulted in more requirements for the storage of electric energy in electronic devices, and thus increasing attention has been paid to improve energy efficiency.^1–3^ Among the major energy storage devices, electrostatic capacitors have an ultrahigh power density and high energy storage efficiency compared to other energy storage devices (*e.g.*, batteries, fuel cells and supercapacitors), and thus play an important role in modern electronic and power systems.^4^ In addition, ultrahigh power density and high operating electric fields are necessary in the application of pulsed power generation, medical defibrillators, and power conditioning.^5–7^ Biaxially oriented polypropylene (BOPP) is one of the most widely used commercial polymer capacitor, which has a high *E*_b_ (∼700 MV m^−1^), ultrahigh discharge efficiency (*η*, >95%), great processability, low cost and long lifetime.^8^ However, its low discharge energy density (*U*_e_) (∼2 J cm^−3^) requires BOPP to have a larger volume for higher discharge energy storage, although it has many advantages in polymer capacitors.^9,10^ In general, for a linear dielectric material, *U*_e_ has the following relationship:

1 *U*_e_ = 1/2*DE* = 1/2*ε*_0_*ε*_r_*E*^2^

where *ε*_0_ and *ε*_r_ are the vacuum permittivity and the effective permittivity of the dielectrics, respectively.^11^ It can be concluded that the main reason for the low *U*_e_ of BOPP is its low intrinsic *ε*_r_. Thus, it is desirable to find polymer materials with simultaneously high *E*_b_ and *ε*_r_ to fabricate dielectric capacitors with ultrahigh *U*_e_.^12^ For polar polymers, such as poly(vinylidene fluoride) (PVDF)-based ferroelectric polymers, they have a higher dielectric constant due to the presence of polar groups and are considered as promising dielectric energy storage materials.^13^ For example, P(VDF-TrFE-CFE) achieves a high *U*_e_ of 10 J cm^−3^ at 350 MV m^−1^ due to its high *ε*_r_ (∼50 at 1 kHz).^14,15^ For another PVDF-based polymer, P(VDF-HFP), it can also achieve a high *U*_e_ of 13.5 J cm^−3^ and *η* of 62% at 600 MV m^−1^.^16^ In order to further explore potential dielectric polymers for energy storage, tremendous works have been carried out to achieve higher *U*_e_, and good results have been obtained.^17,18^ One classic approach is to introduce higher *ε*_r_ nano-inorganic fillers into the polymer matrix and then investigate the dielectric properties of the nanocomposite (such as PVDF/BaTiO_3_ nanocomposite, PVDF/barium strontium titanate (BST) nanocomposite and P(VDF-HFP)/BaTiO_3_@TiO_2_ nanocomposite).^19–24^ Therefore, polymer-based dielectric materials with the advantages of high power density, high breakdown strength (*E*_b_) and excellent flexibility can be perfectly applied in these fields.^25,26^ However, the results showed that the *E*_b_ of nanocomposites significantly decrease with the introduction of inorganic fillers even though *ε*_r_ increases to a certain extent, which is mainly due to the electrical mismatch between ceramic fillers and the polymer matrix.^27,28^ The other method is to design nanocomposite structures by controlling the distribution of fillers that can enhance the *ε*_r_ of the nanocomposite while maintaining relatively high *E*_b_. Specifically, the fiber orientation, interpenetrating gradient, multilayer filler, core–shell structure and array structure can yield higher *U*_e_, and reliable dielectric properties can be obtained from these nanocomposites.^26,27,29–34^ However, all these nanocomposites show a relatively low discharge efficiency, *η* (<75%), which is a critical issue that has long been overlooked in previous studies on dielectric materials.^25,35^ A low discharge efficiency means that a massive amount of wasted energy has to be converted into surface energy of breakdown paths and Joule heat, resulting in early failure of dielectric materials before their intrinsic *E*_b_. Taking PVDF-based nanocomposites as an example, the low *η* primarily originates from (i) ferroelectric loss and (ii) conductance loss.^28,36,37^ The ferroelectric loss is mainly due to the fact that the dipole turnover cannot keep up with the applied alternating electric field, which shows an increase in dielectric loss caused by hysteresis. The dipole size is directly related to the ferroelectric loss, which determines how fast it responds to the electric field and affects the value of the ferroelectric loss.^38^ The conductance loss is caused by the local electric current caused by the movement of carriers inside the material under the action of an electric field.^39^ In contrast, an extremely high discharge efficiency can be found in paraelectric polymers, which belong to a category of dielectric materials without permanent dipolar moments along their chains (*e.g.*, PMMA, PSF, PEI, *etc.*).^40,41^ Recently, multilayer structures have been used to combine high efficiency polymers with high polarization polymers to achieve an enhancement in the dielectric properties of composite materials. Compared with the single layer structure, the multilayer structure can avoid early breakdown of composites *via* its own blocking effect, thus increasing the breakdown strength of composite materials.^42^ In addition, different from the interfacial mismatch problems caused by introducing inorganic particles into a polymer matrix, a better contact interface is formed between polymer multilayers with no inorganic particles added, which can effectively reduce the internal defects of composites and further improve the dielectric properties of composites. Zhu *et al.* prepared a series of multilayer films based on PVDF (such as PC/PVDF, and PSF/PVDF), and the experimental results showed that the multilayer structure well combined the high polarization performance of PVDF with the high breakdown performance of the other component.^43^ Wang *et al.* prepared a series of PMMA/PVDF three-layer composite structures with different PMMA contents and obtained a great improvement in discharge efficiency.^44^ Therefore, the integration of a polar polymer with a non-polar polymer in dielectric materials through the multilayer structure approach provides a promising opportunity towards achieving a high *U*_e_ and high *η* simultaneously.

Herein, we fabricated a series of P(VDF-HFP)/PMMA alternating multilayer composite films, in which P(VDF-HFP) and PMMA were superimposed layer by layer, and the effects of the multilayer structure on the composite dielectric behavior were studied in detail. The results demonstrate that the PMMA layer and the P(VDF-HFP) layer of the composite films have a dense and stable interface, benefitting from their good compatibility. More importantly, the free movement of carriers in the composite films is significantly inhibited by the introduction of high insulation PMMA and multilayer structure, which is manifested in the reduced leakage current of the composite films. Meanwhile, the microstructure of the films transforms into an amorphous phase under the influence of PMMA, and hence they exhibit low ferroelectric loss. The lower leakage current and low ferroelectric loss of the multilayer composite films may not only improve their electrical breakdown strength but also significantly enhance their discharge efficiency. As a result, by combining P(VDF-HFP) with high polarization and PMMA with excellent insulation properties into the multilayer structure, extremely high *η* (∼84%) and ultrahigh *U*_e_ (∼25.3 J cm^−3^) are achieved in multilayer composite films.

## Experimental

### Fabrication of PMMA/P(VDF-HFP) multilayer composite films

The multilayer composite films with PMMA and P(VDF-HFP) were fabricated *via* a non-equilibrium process. Briefly, P(VDF-HFP) powder (Arkema, France, Kynar Flex 2801 with 10 wt% HFP) was firstly thoroughly dissolved in the mixed solvent of *N*,*N*-dimethylmethanamide (DMF) and acetone, and then magnetically stirred for 12 h. PMMA powder was treated the same as the P(VDF-HFP) powder, except it was stirred in a 45 °C water bath. The obtained solutions were then transferred to two different syringes as the electrospinning precursor solutions. Then, a modified electrospinning process was employed to prepare the multilayer composite nonwoven fabric under the conditions of an electric field of 1.0 kV cm^−1^ and a flow rate of 1.0 mL h^−1^. To fabricate multilayer films denoted as M-3L, M-5L, M-7L and M-9L films with the same thickness (∼12 μm), PMMA and P(VDF-HFP) were alternately electrospun onto the same collector, with the total electrospinning time fixed at 70 and 80 min for PMMA and P(VDF-HFP), respectively. The total electrospinning time (70 and 80 min) was equally distributed for all the PMMA and P(VDF-HFP) layers to form an alternating multilayer structure. The collected fabrics were then hot-pressed at 200 °C under a pressure of 10 MPa for 1 h. The as-pressed composite films were annealed at 190 °C for 7 min followed by a quenching process in ice-water. A similar process was also applied to fabricate pure PMMA and pure P(VDF-HFP) films. Therefore, a series of dense nanocomposite films with different composition distributions but the same compositional contents were obtained.

### Characterization

The morphology of the nanofibers and films and the corresponding thickness of the films were characterized *via* scanning electron microscopy (SEM, Zeiss, MERLIN VP Compact). The crystal structures of the nanocomposite films were analysed *via* X-ray diffraction (XRD, Rigaku SmartLab). Furthermore, dielectric spectroscopy on the films was measured using a Novocontrol (Keysight Technologies, Inc.) Alpha-A high-resolution dielectric analyser at room temperature within a wide frequency range of 10^0^ to 10^7^ Hz. Unipolar displacement–electric field (*D*–*E*) hysteresis loops were measured at 10 Hz with a Premier II ferroelectric test system (Radiant Technologies, Inc.). DC leakage current density (in A cm^−2^) measurements were performed using the same ferroelectric test system. The samples were placed in the sample chamber of the thermally stimulated discharge current (TSDC, Novocontrol) for the TSDC study. A DC poling electric field of 15 MV m^−1^ was applied on the nanocomposite films at a 60 °C polarization temperature. After poling for 30 min, the samples were rapidly cooled to −100 °C while maintaining the electric field. After reaching −100 °C, the electric field was removed, and the samples were heated to 100 °C at a heating rate of 5 °C min^−1^. At this time, the depolarization current corresponding to the temperature change was obtained.

## Results and discussion

**Fig. 1:**
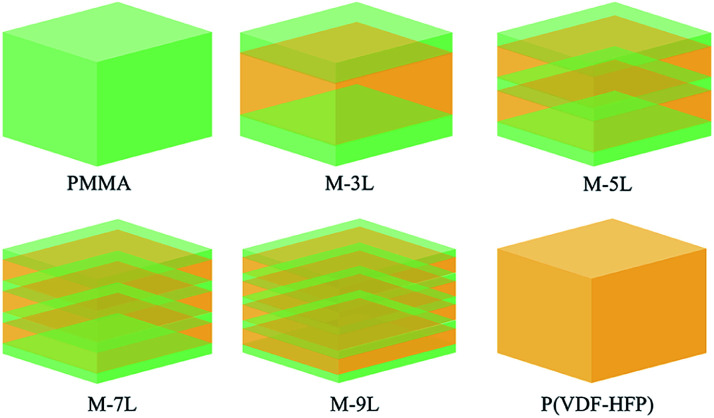
Schematic illustration of the structures of the PMMA/P(VDF-HFP) multilayer composites and the controls (pure PMMA and pure P(VDF-HFP)), respectively.

PMMA/P(VDF-HFP) multilayer composites dielectric capacitors, where the volume fraction of PMMA was fixed at 40 vol%, were fabricated through a non-equilibrium processing method.^45^ Firstly, a modified electrospinning process was applied to prepare PMMA and P(VDF-HFP) nanofibers (∼200 nm). As shown in Fig. S1a and b,† the PMMA nanofibers and pure P(VDF-HFP) nanofibers both have a smooth surface and uniform thickness. Then, through a layer-by-layer process, a multilayer nonwoven fabric was formed due to the alternating superposition of PMMA nanofibers and P(VDF-HFP) nanofibers. After hot-pressing treatment, the nonwoven fabric was transformed into a dense nanocomposite film, and a well-defined layered structure was observed by adding 3 vol% of BaTiO_3_ nanoparticles (not present in subsequent test films) as an internal mark in PMMA, as shown in Fig. S1c–f.† Here, we prepared four types of multilayer composite films with different layer numbers, *i.e.*, 3L, 5L, 7L, and 9L, and the multilayer structures are shown in Fig. 1. Note that the PMMA content of each layer was controlled by the electrospinning time, which was equally distributed with the total PMMA electrospinning time of 70 min. The P(VDF-HFP) layers were obtained under the same electrospinning conditions except the total time was 80 min. Meanwhile, pure PMMA and P(VDF-HFP) films were also prepared *via* the same preparation process for comparison. The as-pressed films also needed to be thermally treated to achieve a controllable crystal phase. As shown by the XRD patterns (Fig. S2†), the introduction of the multilayer structure and the presence of amorphous PMMA led to a gentle amorphous peak in the multilayer film. In addition, it can be found that the angle range of the amorphous peak of the thin film is 10–22, while that of PMMA and P(VDF-HFP) is 8–20 and 15–22, respectively. This phenomenon also reflects the XRD peak superposition. Therefore, the film as a whole is an amorphous phase, which means that the polarization properties and the ferroelectric loss of the materials are reduced.

**Fig. 2:**
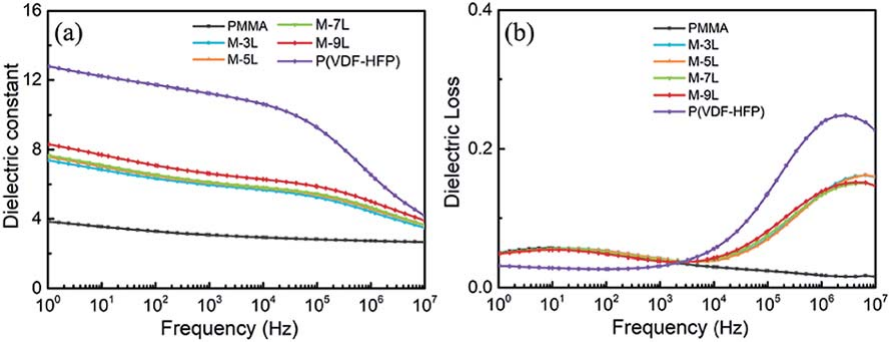
(a) Frequency dependence of dielectric constant and (b) dielectric loss for the pure polymer and multilayer composite films.

To understand the effect of the multilayer structure on the dielectric properties of the composite films, frequency sweep dielectric spectroscopy for the multilayer composite films was conducted and the results are presented in Fig. 2. It can be seen that the dielectric constant of PMMA remained almost linear at around 4 over the frequency range because of its amorphous structure, while for P(VDF-HFP), its dielectric constant has a strong dependence on frequency due to its partial crystalline structure and corresponding polarity. The introduction of the multilayer structure caused a change in the polymer structure, leading to the combination of the dielectric properties of PMMA and P(VDF-HFP) for the composite films. Specifically, the dielectric constants of the multilayer films (M-3L, M-5L, M-7L, and M-9L) are nearly two-fold that of pure PMMA over the full frequency range, which is mainly due to the introduction of P(VDF-HFP), which possesses higher permittivity. Simultaneously, it can be found that with an increase in the number of alternating layers, the dielectric constants of the films slightly improved due to the enhanced influence of interfacial polarization at relatively high frequencies. Fig. 2b compares the dielectric loss of the various films. It can be observed that the introduction of P(VDF-HFP) with a higher dielectric loss inevitably led to an increase in the overall loss of the films. However, significant reductions in dielectric loss were found in the multilayer films at high frequencies. The loss peak at high frequency comes from the α relaxation of the P(VDF-HFP) chain segment.^37^ After combining with PMMA, part of the P(VDF-HFP) chain segments is affected by the PMMA chain segment and the movement of the P(VDF-HFP) chain is restricted, which leads to a reduction in the loss peak of P(VDF-HFP) at high frequency. In the low frequency, a loss peak corresponding to the α relaxation of the PMMA chain segments can also be observed.^46^ When PMMA was combined with P(VDF-HFP) through the multilayer structure, since PMMA still exists alone, a corresponding loss peak still existed in the multilayer film at low frequency. As a result, composite films with low loss and high dielectric constant were achieved *via* the combination of PMMA and P(VDF-HFP), providing a good basis for the subsequent improvement of related dielectric properties, which are highly required for energy storage.

**Fig. 3:**
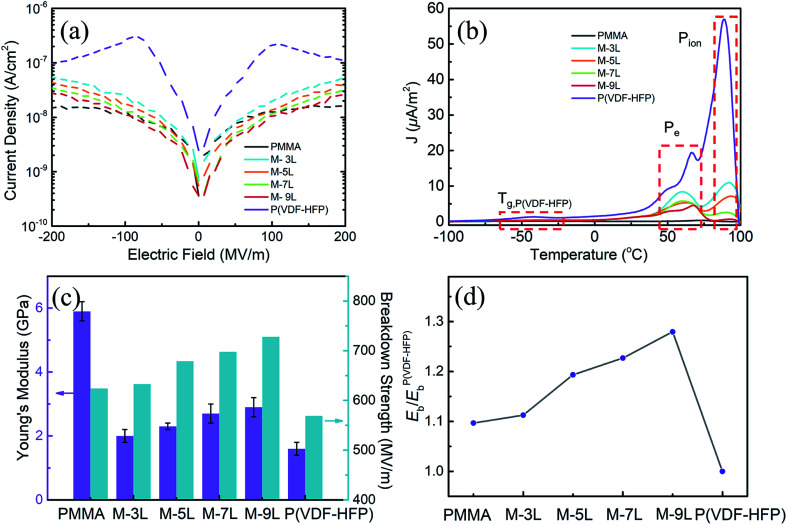
(a) Variation in leakage current *vs.* electric field and (b) TSDC spectra for the pure polymer and multilayer composite films. (c) Young's modulus and breakdown strength for the pure polymer and multilayer composite films. (d) Comparison of the experimental breakdown strength of the multilayer composites films.

For dielectric materials, in addition to excellent dielectric properties, high breakdown strength is another important parameter to determine the quality of the materials. Controlling low conduction loss is an important way to improve the breakdown strength of dielectric materials. Fig. 3a shows the electric field dependence of the leakage current for the PMMA/P(VDF-HFP) multilayer composites with an increasing number of layers. The pure PMMA and the composite films with a multilayer structure have dramatically lower leakage currents than pure P(VDF-HFP). This result indicates that the multilayer structure can effectively suppress the leakage current, and the suppression effect is better with an increase in the number of layers. In general, the leakage current is mainly caused by the movement of charge carriers, which are originally inside the films or injected from the electrodes before the film is broken down. Therefore, the reduction in the leakage current of the nanocomposite films is mainly contributed by the following two factors.^47^ Firstly, the leakage current of the film can be well controlled at a lower level due to the excellent insulation properties of PMMA. Secondly, with the introduction of the multilayer structure, the free motion of carriers can be restrained at the interface between PMMA and P(VDF-HFP) by taking advantage of the different electrical performances between them.

To further analyze the contribution of the multilayer structure in suppressing the leakage current, TSDC spectroscopy was used to study the polarization of various carriers (such as electrons and impurity ions) in multilayer composite films. Fig. 3b shows the TSDC spectra of the multilayer films with a poling electric field (*E*_p_) of 15 MV m^−1^ and a poling temperature (*T*_p_) of 60 °C for 30 min. The polarization conditions of pure PMMA and P(VDF-HFP) were kept the same as that of the multilayer composite films. For the TSDC spectroscopy of pure P(VDF-HFP), three discharge peaks could be identified at around −45 °C, 60 °C, and 90–100 °C. Among them, the peak at −45 °C (*T*_g,P(VDF-HFP)_) corresponds to the depolarization of the polarized amorphous P(VDF-HFP) *via* devitrification at *T*_g_ (glass transition temperature), the peak of 60 °C (*P*_e_) corresponds to the depolarization of the injected charges from electrodes (from Schottky or thermionic emission), and the discharge peak at around 90–100 °C (*P*_ion_) can be assigned to the depolarization of ions in the P(VDF-HFP) film.^48^ The difference in the peak position between *P*_e_ and *P*_ion_ is because the ions need to be polarized at a higher poling temperature since their mobility is lower than the electron mobility. Conversely, for PMMA under the same test conditions, there is almost no corresponding depolarization peak at the temperature range of −100–100 °C, which also proves that PMMA has excellent insulation performance. By comparing the depolarization peaks of the different films in Fig. 3b, it can be found that the intensity of all the depolarization peaks was significantly weaker for the multilayer composite films compared with that of P(VDF-HFP), and it became weaker as the number of layers increased. For example, the peak intensity of P_ion_ in the P(VDF-HFP) film is ∼56 μA m^−2^ while it is only ∼0.78 μA m^−2^ for the 9L multilayer films. One of the primary reasons for the decrease in intensity is the excellent insulation properties and high *T*_g_ of PMMA. Firstly, when PMMA is added to P(VDF-HFP), the movement of the P(VDF-HFP) chain is inevitably affected by the PMMA chain segment, and the local electric field of P(VDF-HFP) with a high dielectric constant is smaller according to the multilayer theory, causing weaker *T*_g,P(VDF-HFP)_ peaks. Simultaneously, the high insulation of PMMA can well suppress the free movement of carriers in the films and improve their overall insulation performance. The introduction of the multilayer structure is another main reason for the decrease in the intensity of the peaks, mainly reflected in the reduction of *P*_e_ and *P*_ion_. As mentioned above, the peaks of *P*_e_ and *P*_ion_ are formed by the slow release of thermally stimulated carriers during the heating process, and the magnitude of the peak intensity directly reflects the number of freely movable carriers. Thus, it can be concluded that the multilayer structure can reduce the number of freely movable carriers, and the reduction effect enhances as the number of stacked layers increases. The reason for this is that the thermally stimulated carriers move along the direction of the electric field, and due to the multilayer structure, most of the carriers are confined at the interface between PMMA and P(VDF-HFP). Simultaneously, these trapped carriers cannot be completely released during the subsequent heating process due to the interface confinement, and finally the peaks of *P*_e_ and *P*_ion_ are effectively reduced.

In addition to the electrical breakdown caused by leakage conduction, the electromechanical breakdown under a high electric field is also a major reason for the breakdown of the composite films. Theoretically, the Stalk–Garton model gives the relationship between the breakdown electric field and Young's modulus as: *E*_em_ = *κ*(*Y*/*ε*_0_*ε*_r_)^1/2^ (*κ* is a constant and *Y* is the Young's modulus), which visually shows that these two factors are positively correlated.^49^ During the experiment, when a high electric field is applied in the out-of-plane direction of the nanocomposite films, the breakdown resistance of the multilayer composite films is largely dependent on the out-of-plane mechanical properties. The Young's modulus of the multilayer composite films in the out-of-plane direction was evaluated by nanoindentation and presented in Fig. 3c. As can be seen, the Young's modulus of pure P(VDF-HFP) is only 1.6 GPa, which is much lower than that of pure PMMA (5.8 GPa). By introducing the hard PMMA and multilayer structure into P(VDF-HFP), the Young's modulus of the multilayer composite films improved compared with that of the pristine P(VDF-HFP). The results also show that the Young's modulus of the multilayer composite films with the same composition increased with an increase in the number of layers. For the multilayer composite films, the increase in the number of layers with the same composition indicates a larger interaction between PMMA and P(VDF-HFP), which can better enhance the mechanical properties of the multilayer composite films.

Benefitting from the reduction in leakage current and the enhancement in the mechanical properties of the multilayer composite films, their breakdown strength significantly improved compared to that of P(VDF-HFP). Two-parameter Weibull statistics was employed for the analysis of the dielectric breakdown behavior of the multilayer composite films. The distribution function is described as: *P*(*E*) = 1 − exp[−(*E*/*E*_b_)^*β*^], where *P*(*E*) is the cumulative probability of electric failure, *E* is the experimental breakdown strength, *E*_b_ is calculated from the Weibull distribution, which refers to the breakdown strength at the cumulative failure probability of 63.2% and is also regarded as the characteristic breakdown strength, and *β* is a shape parameter to assess the degree of data dispersion and is also an important parameter for evaluate the quality of the multilayer films.^7^ The Weibull statistical analysis for the multilayer films is shown in Fig. S3,† and the values of *E*_b_ are summarized and plotted in Fig. 3c. Clearly, it can be found that *E*_b_ was greatly enhanced with an increase in the number of layers, from 569 MV m^−1^ for the pure P(VDF-HFP), to a maximum of 728 MV m^−1^ for the nanocomposite with 9 layers, which is nearly 1.3 times that of pristine P(VDF-HFP). To further study the impact of the multilayer structure on *E*_b_, the *E*_b_ of multilayer film and PMMA were compared with that of P(VDF-HFP), and the calculation results are presented in Fig. 3d. From the calculation results, two distinct features can be distinguished in the dielectric breakdown behaviors of the multilayer composites films. Firstly, it can be concluded that the multilayer structure can significantly improve the *E*_b_ of the composite films, and the key factor that affects the breakdown of the nanocomposite films with the same composition is the number of layers. For instance, the four multilayer nanocomposites with M-3L (633 MV m^−1^), M-5L (679 MV m^−1^), M-7L (698 MV m^−1^) and M-9L (728 MV m^−1^) configurations showed higher breakdown strengths than P(VDF-HFP) (569 MV m^−1^). Secondly, through the previous calculation results, it can be found that different from the simple addition effect, the *E*_b_ of the multilayer composite films is not only higher than that of P(VDF-HFP), but also pure PMMA. This means that in addition to the reduction in leakage conduction and the improvement in electromechanical performance caused by the multilayer structure, the blocking effect of the interface is particularly important for the improvement of breakdown strength. Meanwhile, all the multilayer composites films exhibit rather high *β* values (∼20), which are significantly higher than that of PMMA (19) and P(VDF-HFP) (11), suggesting that the *E*_b_ distribution is narrow and the reliability of the film was improved significantly. The high *β* values also provide an important basis for demonstrating the excellent dielectric properties of multilayer composite films.

**Fig. 4:**
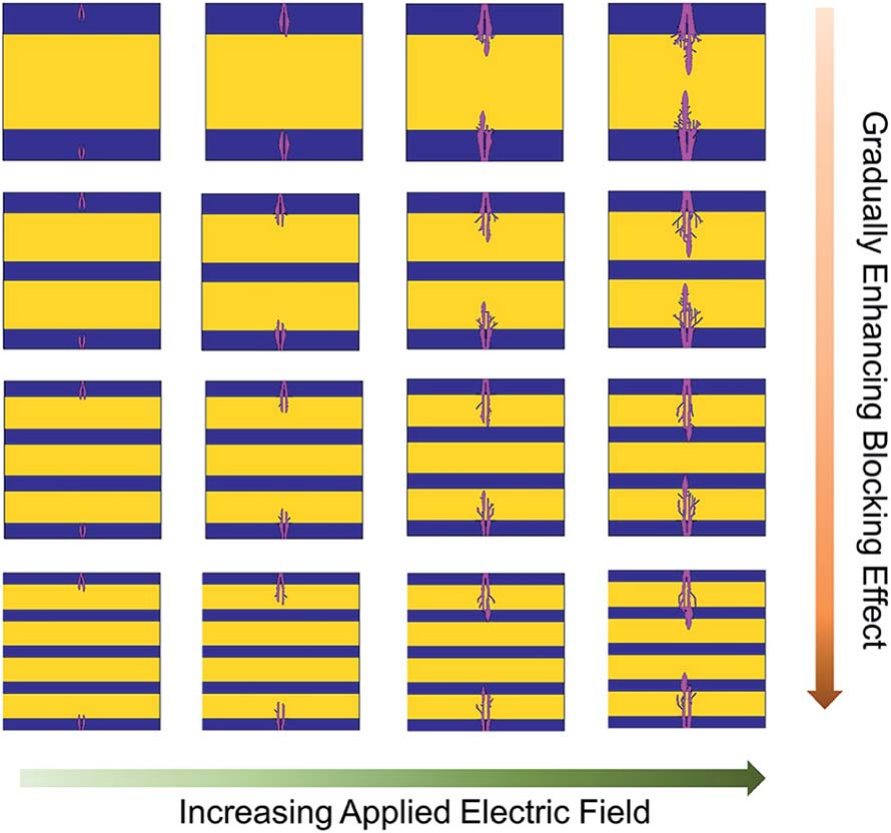
Breakdown evolution procedures for the multilayer composite films with 3L, 5L, 7L and 9L by phase-field simulation.

To further study the influence of the interface on the electric breakdown process in the multilayer composite films, a phase-field model was employed to simulate the growth of electrical trees in the film with an increase in time under an electric field, and the contribution of the interface blocking effect to the breakdown strength is discussed. In this case, we constructed four multilayer structure models, as shown in Fig. 4. As can be seen, the electrical trees of all the multilayer composite films increased with strengthening of the electric field, but with an increase in the number of layers, the longitudinal growth and lateral expansion of the electrical trees were inhibited. The interface blocking effect can be used to explain this phenomenon, which includes two aspects. Firstly, there are many deep traps at the interface between PMMA and P(VDF-HFP), which capture some charge carriers and confine them in the interface region, reducing the breakdown possibility of the nanocomposite films caused by carrier motion. Hence, it is reasonable to infer that the greater the number of layers, the greater the amount of carriers restricted, and the reliability of the nanocomposite films will be better improved. Secondly, according to the theory of partial voltage, when the film is subjected to an electric field, PMMA is assigned a voltage higher than P(VDF-HFP) due to the dielectric mismatch between PMMA (*ε*_r_ ∼ 3) and P(VDF-HFP) (*ε*_r_ ∼ 9). Therefore, the P(VDF-HFP) layer with a low *E*_b_ in the multilayer structure can avoid early failure under the higher electric field, which is also a reason for the breakdown strength improvement. Actually, the interface blocking effect means that the electrical breakdown path is blocked, and the multilayer structure makes use of this advantage to improve the breakdown strength to a higher value.

**Fig. 5:**
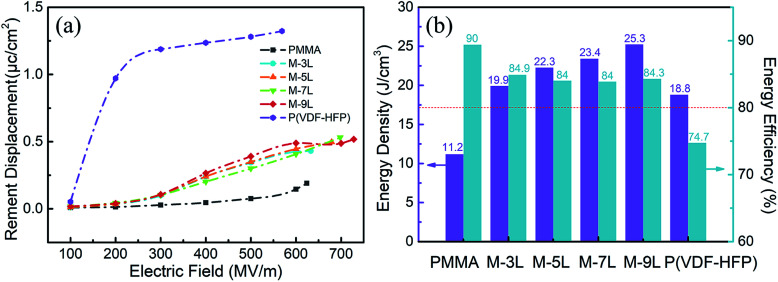
(a) Remnant displacement of the multilayer composite films as a function of the electric field summarized from the D-E loops in Fig. S4.† (b) Comparison of the energy storage properties of the multilayer composite films.

The electrical displacement–electrical field (*D*–*E*) loops of the pure polymer and multilayer composite films at high electric field were measured using a modified Sawyer–Tower circuit. Fig. S4† shows the *D*–*E* loops for all the dielectric films. PMMA exhibited an extremely thin line type, indicating a very low dielectric loss, while P(VDF-HFP) as a polar polymer presented a coarser line, indicating a high dielectric loss. In addition, the *D*–*E* loops of the multilayer composite films, which combine PMMA and P(VDF-HFP) through the stack method, were significantly narrow compared with that of pure P(VDF-HFP) due to the addition of the linear PMMA, and the maximum polarization values also decreased accordingly. Fig. 5a compares the remnant polarization of the various films derived from their *D*–*E* Loops (Fig. S4†) as a function of electric field. The Pr of P(VDF-HFP) increased rapidly with an increase in the electric field from 0.053 μC cm^−2^ at 100 MV m^−1^ to 1.188 μC cm^−2^ at 300 MV m^−1^, and then very mildly to 1.323 μC cm^−2^ at 569 MV m^−1^. This exhibits a stark contrast to the Pr changing trends of the multilayer composite films, which increased monotonically with an increase in electric field, and especially the pure PMMA, its Pr had almost no change. The variation in Pr at lower electric fields is primarily associated with the intrinsic leakage current of the material. Combined with the leakage current of the composite films at a lower field presented in Fig. 3a, the results are also consistent with the above conclusions. Therefore, it can be concluded that the rapid growth of Pr for P(VDF-HFP) can be effectively suppressed by introducing PMMA with an excellent insulation performance, while the blocking effect of the multilayer structure can also provide positive effects to reduce the Pr of the P(VDF-HFP) nanocomposites.

According to formula (1), the multilayer composite films will inevitably induce a higher discharge energy density with a decrease in Pr and increase in *E*_b_. Fig. S5† shows the *U*_e_ and *η* of the dielectric films as a function of electric field, both of which are related to the integration of the area between the discharge/charge curve and the ordinate in the *D*–*E* loops. As shown in the graph, even the pure PMMA has an extremely high discharge efficiency before breakdown, where its energy density is relatively low due to its low polarization value, while the opposite is observed for P(VDF-HFP). For the films with the multilayer structure, owing to the enhanced *E*_b_ induced by the multilayer structure, *U*_e_ is remarkably increased compared with that of PMMA and P(VDF-HFP), and a higher efficiency is obtained, benefitting from the suppressed dielectric loss. For a better comparison, the values of *U*_e_ and *η* are summarized and plotted in Fig. 5b and two significant results can be observed. Firstly, the energy densities of all the multilayer films are almost higher than 20 J cm^−3^, which is an ultrahigh value for polymer matrix dielectric materials. For instance, the highest *U*_e_ of ∼25.3 J cm^−3^ at 728 MV m^−1^ was obtained for M-9L, which is 1.34 times that of P(VDF-HFP) (*U*_e_ ∼ 18.8J cm^−3^ at 569 MV m^−1^) and 2.25 times that of PMMA (*U*_e_ ∼ 11.2 J cm^−3^ at 616 MV m^−1^). The *U*_e_ of 3L (∼19.9 J cm^−3^ at 633 MV m^−1^), 5L (∼22.3 J cm^−3^ at 679 MV m^−1^) and 7L (∼23.4 J cm^−3^ at 698 MV m^−1^) are also higher than that of P(VDF-HFP) and PMMA. For polymer matrix composites, the contribution of *E*_b_ dominates that of the dielectric constant to *U*_e_, which is due to the fact that the dielectric constant of polymer composites is generally at a lower level and has a first power relationship with *U*_e_. The second characteristic is the enormous improvement of *η*. The extremely high efficiency means that most of the energy can be effectively used and only a very small amount of the energy is converted into Joule heat or otherwise during energy storage and release. For P(VDF-HFP), an *η* value of 74.7% was achieved through the optimization of the preparation process, which is still unsatisfactory to meet application requirements. Inspiringly, an ultrahigh *η* of 84% and *U*_e_ value of 25.3 J cm^−3^ (M-9L) can be acquired from the P(VDF-HFP)-based multilayer films with PMMA, which are attributed to the suppression of ferroelectric loss and conduction loss of P(VDF-HFP).

## Conclusions

In summary, a series of PMMA and P(VDF-HFP) multilayer composite films with different numbers of layers were prepared by alternating superposition, where a stable and dense interface existed between PMMA and P(VDF-HFP), benefitting from their good compatibility. By inserting the nonpolar PMMA into the layers of the polar P(VDF-HFP), the properties of the multilayer composite films showed large contrasts compared with pure P(VDF-HFP). The high insulation of PMMA plays a key role in reducing the leakage current of the composite films. Meanwhile, the multilayer structure can reduce the number of carriers and suppress their movement. This synergistic effect increased with an increase in the number of layers, which was caused by the interface blocking effect. Thus, the multilayer composite film achieved a substantial increase in the breakdown strength, especially for the *E*_b_ of M-9L to an outstanding 728 MV m^−1^, which is even higher than that of the pristine PMMA. On the other hand, the presence of PMMA with low ferroelectric loss has an effect on the motion of the P(VDF-HFP) segments, which suppresses the high ferroelectric loss of P(VDF-HFP). As a result, a significant improvement in *η* (∼84%) and *U*_e_ (>20 J cm^−3^) of these films was achieved through the multilayer structure. We suggest that this all-organic multilayer structure without the presence of an inorganic and organic interface can realize a stable and dense interface, and the outstanding advantage of this structure is to improve the breakdown performance of films. Therefore, this work provides an effective and simple way to design dielectric capacitors with high reliability and high discharge efficiency under a high electric field with low sacrifice of the energy density.

## Conflicts of interest

There are no conflicts to declare.

## Supplementary Material

RA-010-C9RA10030J-s001
