# Supplementary material for: An alternating multilayer architecture boosts ultrahigh energy density and high discharge efficiency in polymer composites
Source: RSC Adv. 2020 Feb 6;10(10):5886–93. doi: 10.1039/c9ra10030j (PMC9049627; doi:10.1039/c9ra10030j)
Supplement: RA-010-C9RA10030J-s001 [file RA-010-C9RA10030J-s001.pdf]

Electronic Supplementary Material (ESI) for RSC Advances.
This journal is © The Royal Society of Chemistry 2020

Supporting Information

Alternating Multilayer Architecture Boosts Ultrahigh Energy Density and High Discharge Efficiency in Polymers Composites

Tao Zhang, [a] Zhenkang Dan, [a] Zhonghui Shen, [a] Jianyong Jiang, [a] Mengfan Guo, [a] Bin Chen, [*a] Yuanhua Lin, [a] Ce-Wen Nan, [a] Yang Shen[*a]

a School of Materials Science and Engineering, and State Key Lab of New Ceramics and Fine Processing, Tsinghua University, Beijing 100084, China. E-mail: shyang_mse@tsinghua.edu.cn ; chenbinalvin@mail.tsinghua.edu.cn

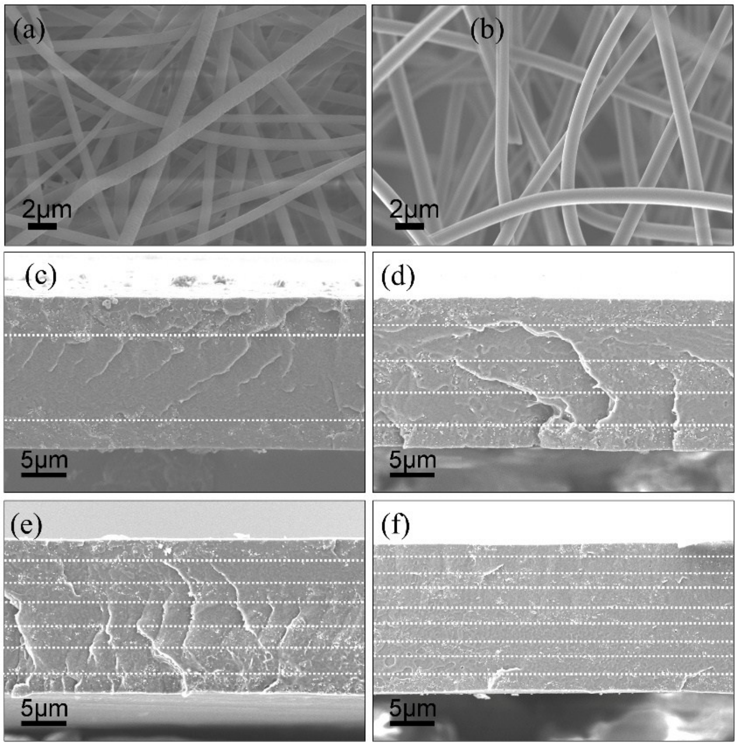

Figure S1. Fiber SEM images of the (a) Pure PMMA and (b) Pure P(VDF-HFP). SEM images of the PMMA/P(VDF-HFP)-BTO_nps multilayer composite films with 3L (c), 5L (d) 7L (e) and 9L (f), respectively.

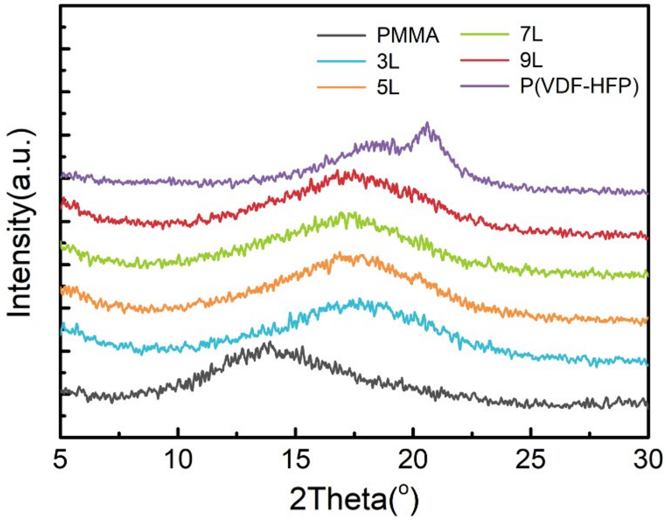

Figure S2. XRD patterns of the multilayer composite films.

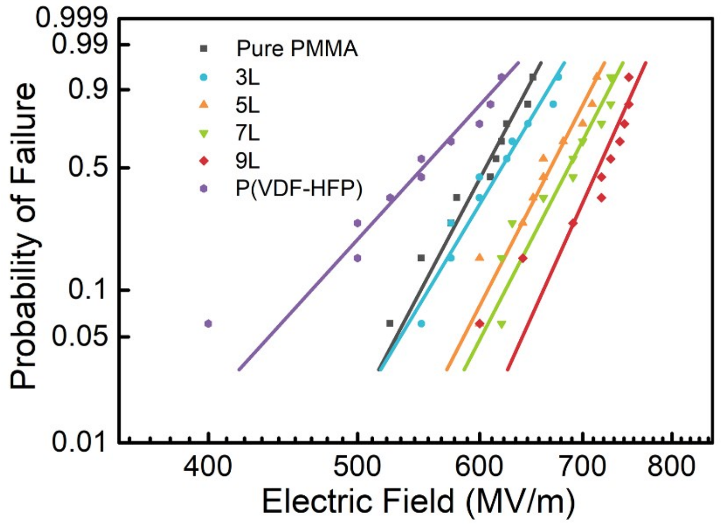

Figure S3. Weibull distributions of electric breakdown strength at room temperature for multilayer composite films.

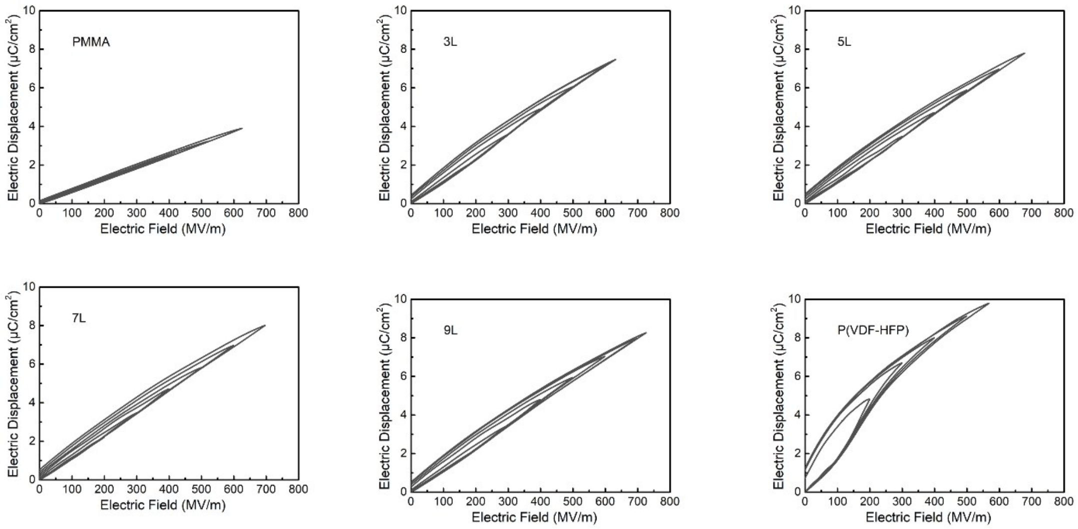

Figure S4. Unipolar electric displacement-electric field (*D*-*E*) loops for multilayer composite films.

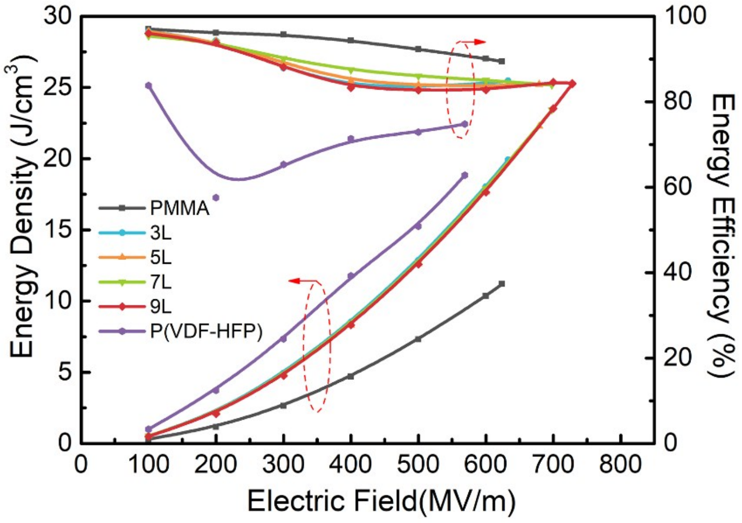

Figure S5. Discharged energy density and charge–discharge efficiency of the multilayer composite films as a function of the electric field.
